# Synergistic Antioxidant Effects of Molecular Hydrogen and Cold Atmospheric Plasma in Enhancing Mesenchymal Stem Cell Therapy

**DOI:** 10.3390/antiox13121584

**Published:** 2024-12-23

**Authors:** Mikhail Yu. Artamonov, Felix A. Pyatakovich, Inessa A. Minenko

**Affiliations:** 1 Department of Physical Medicine and Rehabilitation, Penn Medicine Princeton Health, Plainsboro, NJ 08536, USA; 2 Department of Internal Medicine, Belgorod State University, 308015 Belgorod, Russia; piatakovich@bsu.edu.ru; 3 Department of Rehabilitation, Sechenov Medical University, 119991 Moscow, Russia; minenko_i_a@staff.sechenov.ru

**Keywords:** mesenchymal stem cells, molecular hydrogen, cold atmospheric plasma, oxidative stress, regenerative medicine

## Abstract

In regenerative medicine, mesenchymal stem cells (MSCs) have shown their importance and potential in tissue reconstruction and immune system modification. However, such cells’ potential is often diminished by factors such as oxidative stress, immune rejection, and inadequate engraftment. This review highlights the role of molecular hydrogen (H_2_) and cold atmospheric plasma (CAP) as adjunct therapies to improve the effectiveness of MSC therapy. H_2_ has strong antioxidative and anti-inflammatory actions as it quenches reactive oxygen species and positively stimulates the Nrf2 pathway that promotes MSC survival and life. CAP, being a modulated source of ROS and RNS, also assists MSCs by altering the cellular redox balance, thus facilitating cellular adaptation, migration, and differentiation. H_2_ and CAP in conjunction with each other assist in establishing an ambience favorable for promoting MSCs’ survival and growth abilities, and reduce the healing time in various pathways such as wound, neuroprotection, and ischemia. Besides these concerns, this review also covers the best administration routes and doses of H_2_ and CAP together with MSCs in therapy. This study informs on a novel dual method aimed at improving the outcome of MSC therapy while adding several molecular targets and relevant clinical uses concerning these therapies. Research of the future has to deal with bettering these protocols so that the therapeutic benefits can be maximized without long-term implications for clinical applications.

## 1. Introduction

Mesenchymal stromal cells (MSCs) can treat several disorders due to their immunomodulatory and regenerative properties. However, many difficulties hinder their clinical implementation and efficacy. MSC treatments seldom meet the main efficacy criteria in clinical trials because they are less effective in people than in preclinical studies. This is mostly owing to cell-based therapy translation variability. The lack of uniform MSC identity criteria across trials causes treatment discrepancies. MSC-based therapy is difficult to standardize and replicate due to this inconsistency [1].

**Figure 1:**
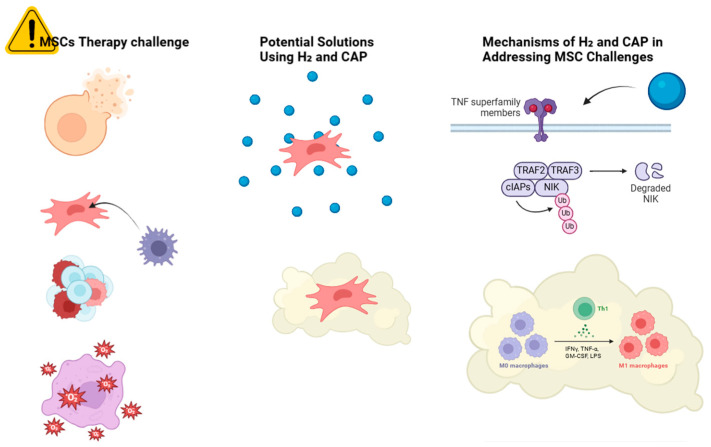
Schematic overview of the challenges in MSC therapy and potential solutions using H_2_ and CAP. The challenges include a low survival rate, immunogenicity, tumorigenicity, oxidative stress, and inflammation. followed by H_2_ solution, they have improved cell survival, antioxidant protection, and reduced cellular mutations. Similarly, CAP treatment includes immunomodulation.

Allogeneic MSCs may lose their benefits due to immune rejection. Senescence may reduce the therapeutic efficacy of autologous MSCs from elderly people [2]. Delivering and targeting MSCs to specific tissues remains difficult. Cell surface adhesion receptor deficiency often reduces MSC engraftment efficiency and therapeutic efficacy [3]. MSC therapy can be compromised by in vitro and in vivo microenvironments. These factors affect MSC migration, viability, and function [4]. Mesenchymal stem cells (MSCs) can start and advance tumors, making their therapeutic usage risky [5], as shown in Figure 1.

Oxidative stress—an imbalance between ROS production and antioxidant defense—affects stem cell survival, differentiation, and function. This synthesis uses multiple studies to analyze how oxidative stress impacts stem cells. Increased ROS levels inhibit MSC proliferation, promote senescence, decrease osteogenic differentiation, and increase adipogenic differentiation [6]. Damage to cellular macromolecules by oxidative stress causes senescence and apoptosis in stem cells [7]. Oxidative stress inhibits stem cell self-renewal and promotes neural lineage differentiation in human embryonic stem cells. MAPK/ERK1/2 signaling promotes spontaneous neuronal differentiation via reactive oxygen species-induced oxidative stress [8]. Hypoxia preconditioning (HPC) maintains oxidant status and reduces ROS to protect MSCs from oxidative stress. Human adipose-derived stem cells (hASCs) live longer after preconditioning with low H_2_O_2_ concentrations due to increased antioxidant defenses and metabolic changes [8]. The HSP90/NF-κB signaling pathway is essential for neural stem cell (NSC) survival during oxidative stress. Regulating HSP90 can protect against oxidative damage. Oxidative stress triggers stem cell autophagy via the ERK1/2 signaling pathway, which can kill cells if not handled [9].

Antioxidants neutralize free radicals to reduce oxidative stress illnesses. New antioxidant delivery methods and technology have been the focus of recent studies. Cell-based tests, notably those using Caco-2 cells, are becoming more popular for assessing antioxidant activity and bioavailability than in vitro and in vivo methods [10]. Nanoparticles, liposomes, and gel-based formulations are being studied to improve antioxidant bioavailability and efficacy. These techniques overcome deficiencies including dietary antioxidant solubility and instability. Metal-containing catalytic antioxidants, including manganese-based compounds, have shown promise in scavenging a wide range of reactive oxygen species. These are being investigated for their potential in treating cardiovascular, neurodegenerative, and inflammatory diseases [11]. New strategies are being developed to target specific subcellular regions where redox dysregulation occurs, such as mitochondria and caveolae.

These techniques include gene and miRNA therapies, nanoparticle technology, and micro-peptide targeting. Synthesis of antioxidant polymers from sustainable and natural monomers is improving. These polymers are used in food packaging, medicine delivery, and synthetic polymer biodegradation [11]. Endophytes from medicinal plants are being explored as sources of novel antioxidants. These microorganisms produce unique metabolites with antioxidant properties, offering potential for new natural antioxidant drugs.

This study aims to investigate the impact of molecular hydrogen and cold atmospheric plasma on the viability and therapeutic efficacy of mesenchymal stem cells (MSCs). This involves examining the impact of these factors on the antioxidant responses of cells, their biological functions, and their ability to migrate to regenerative areas. This research aims to determine the optimal conditions for maximizing synergistic effects to improve the outcomes of MSC therapy, particularly in the contexts of wound healing and tissue regeneration. Understanding the mechanisms underlying these effects may provide insights for developing more effective treatments for various disorders, including the potential for targeted stimulation of apoptosis.

The research gap specifically pertains to the detailed mechanisms explaining how molecular hydrogen and cold atmospheric plasma (CAP) synergistically enhance antioxidant effects in mesenchymal stem cells (MSCs). This study examines the long-term impacts of combined molecular hydrogen and cold atmospheric plasma treatment on mesenchymal stem cells, with a focus on the potential cytotoxicity and genetic stability, and moreover, evaluates the long-term sustainability of the antioxidant effects. The optimal dosage and administration protocols for combined treatment aim to maximize therapeutic benefits and minimize adverse effects.

## 2. Molecular Hydrogen and Cold Atmospheric Plasma: Fundamental Concepts

Molecular hydrogen (H_2_) and cold atmospheric plasma (CAP) are two separate entities characterized by unique chemical and physical properties. CAP is an ionized gas near room temperature, consisting of neutral particles, charged particles, reactive species, and electrons. It can be produced in ambient air and generates energetic species including electrons, metastables, reactive oxygen species (ROS), reactive nitrogen species (RNS), ultraviolet radiation, and localized electric fields [12]. It is devoid of color, scentless, non-toxic, and extremely combustible. Similarly, CAP can interact with liquids, such as tap water, altering their chemical composition by generating reactive species like hydrogen peroxide, hydroxide ions, and nitrate ions. While these reactive species can be transient, their effects on the liquid’s chemistry can be measured and analyzed using various analytical methods [13].

Hydrogen is the lightest and most prevalent element in the universe. It exists as a gas at standard temperature and pressure, with a boiling point of −252.87 °C. Nonetheless, CAP therapy can elicit substantial biological responses, such as the production of hydrogen peroxide in cancer cells, resulting in DNA and mitochondrial damage, elevated intracellular reactive oxygen species, and the initiation of apoptotic processes. Its effects are being investigated for potential applications in cancer therapy [14]. Although H_2_ is comparatively stable, it can combine with oxygen to produce water, thus releasing energy. It can also engage in many chemical reactions, including hydrogenation and reduction activities.

### 2.1. Generation Methods and Delivery Systems

CAP is an ionized gas generated at near-ambient temperatures, abundant in reactive oxygen and nitrogen species (RONS) including hydrogen peroxide and nitrites [14]. Helium, air, and argon are commonly utilized gases for CAP generation, with plasma jets being the predominant production technique. The most prevalent way of applying CAP to cells or tissues involves the direct use of plasma jets or plasma-treated media [15]. CAP may alter the stem cell niche or directly irradiate stem cells to affect their fate, encompassing adhesion, proliferation, differentiation, and death. CAP-treated hydrogels have been engineered to localize and administer RONS, assuring prolonged release while reducing systemic diffusion [16]. CAP elicits cellular reactions including apoptosis, diminished cell viability, and mitochondrial impairment via the production of reactive oxygen and nitrogen species (RONS). Additionally, CAP can augment drug delivery by enhancing cell membrane permeability, typically necessitating a synergy of plasma-induced electric fields and plasma chemistry.

Delivery mechanisms for molecular hydrogen in MSC therapy generally encompass inhalation, hydrogen-enriched water, or direct injection into the circulatory system. Numerous studies have investigated these strategies to guarantee safe and effective administration. Molecular hydrogen can be delivered via inhalation, consumption of hydrogen-rich water, or intravenous injection of hydrogen-rich saline [17]. These methods have demonstrated an enhancement of the hydrogen concentration in the blood and tissues, which is essential for its therapeutic benefits. Moreover, the inhalation of hydrogen gas has been advocated in cli [18] nical environments for the management of COVID-19 pneumonia, owing to its antioxidative, anti-inflammatory, and anti-apoptotic characteristics [19].

### 2.2. Biological Interactions

The potential biomedical uses of cold atmospheric plasma (CAP) are being investigated more and more, especially because of its capacity to produce reactive oxygen and nitrogen species (RONS). This synthesis examines the connections between CAP and mesenchymal cells, which are essential for numerous therapeutic applications. By combining CAP exposure with certain biomaterials, like those that include silica nanoparticles laden with iron oxide, mesenchymal stem cells (MSCs) can proliferate more quickly and improve osteogenic differentiation [20].

Even cancer cells that have undergone the epithelial-to-mesenchymal transition (EMT) are susceptible to the selective lethal effects of CAP and plasma-activated medium (PAM). This selectivity is ascribed to elevated amounts of reactive oxygen species (ROS) in mesenchymal-like cancer cells [21]. Human skin fibroblasts and adipose-derived stromal cells (ASC) may exhibit a senescence phenotype following brief exposure to CAP. This phenotype is marked by DNA damage, proliferation inhibition, and the release of pro-inflammatory cytokines. Notwithstanding this, the cells preserve certain functional features. CAP-induced reactive oxygen and nitrogen species (RONS) can engage with cellular components to activate signaling pathways, such as the Trk/Ras/ERK pathway, resulting in distinct physiological consequences, including brain development. This underscores the intricate interaction between CAP-generated species and cellular signaling pathways [22]. CAP may alter the microenvironment, resulting in indirect effects on cellular behavior. Modifications in the redox status of the microenvironment can affect cellular viability and apoptotic pathways.

Molecular hydrogen has demonstrated considerable molecular and cellular effects that position it as a potentially beneficial agent in regenerative medicine, particularly regarding its influence on stem cells [23]. A similar gaseous molecule called hydrogen sulfide (H_2_S) can affect the mesenchymal–epithelial transition (MET) in cancer cells and participate in cellular signaling. This could have consequences for interpreting hydrogen’s broader function in cellular processes as summarized in Table 1. 

## 3. Antioxidant Mechanisms

Molecular hydrogen (H_2_) demonstrates substantial antioxidant properties, chiefly via direct scavenging actions, activation of the Nrf2 pathway, and regulation of mitochondrial function. These methods enhance its therapeutic efficacy in addressing disorders associated with oxidative stress. Molecular hydrogen (H_2_) serves as a scavenger for free radicals and reactive oxygen species (ROS), mitigating the detrimental effects of hydroxyl radicals and peroxynitrite while preserving functionally significant ROS [24]. They serve as anti-inflammatory and anti-apoptotic agents by interacting with potent oxidants, such as hydroxyl and nitrosyl radicals, within cells, and mitigating oxidative stress. H_2_ promotes the Nrf2 pathway, an essential regulator of the antioxidant response, by preventing its degradation through KEAP1, further facilitating Nrf2’s translocation to the nucleus to commence the production of antioxidant genes, as found by Liam Baird and Masayuki Yamamoto (2020). By promoting mitophagy, increasing the number of mitochondria, and lowering reactive oxygen species (ROS) levels in the mitochondria, molecular hydrogen (H_2_) improves the survival and myogenic differentiation of adipose-derived stem cells [18].

## 4. Synergistic Effects on MSC Biology

### 4.1. Cell Survival and Proliferation

Mesenchymal stem cells differentiate into many cell types, including osteocytes, adipocytes, chondrocytes, neurons, cardiomyocytes, and endothelial cells, making them promising for application in regenerative treatment [25]. Multiple signals from the cellular and non-cellular environment of the cells control self-renewal in the stem cell-like state, including proliferation, differentiation, and migration [26]. Obtaining enough cells to start cell therapy is a limiting factor in therapeutic applications. Long-term in vitro cell culture to achieve appropriate cell numbers may affect the gene regulation and differentiation capacity of these cells as a result of long-term cell culture-induced stress. Furthermore, cell death following in vivo injection of MSCs is a limiting factor, since the majority of donor MSCs are eliminated after injection and do not engraft in substantial numbers in the recipient system [27]. This demands a more efficient cell expansion mechanism that encourages high cell proliferation, survival, and differentiation. The non-cellular microenvironment can be modified to drive cell proliferation, survival, or differentiation. Several investigations on the non-cellular microenvironment have found that cell shape is an important factor [28]. Other studies have shown that matrix rigidity [29], combined with the mechanical feedback provided by the extracellular matrix proteins, guided MSC differentiation [30]. According to a recent study, MSCs received biochemical signals for differentiation through early extracellular matrix proteins before they were changed by the cells during differentiation [31]. All of these studies strongly show that the non-cellular physical microenvironment affects MSC differentiation, which can be altered for targeted cell differentiation during tissue engineering.

### 4.2. Differentiation Capacity

**Figure 2:**
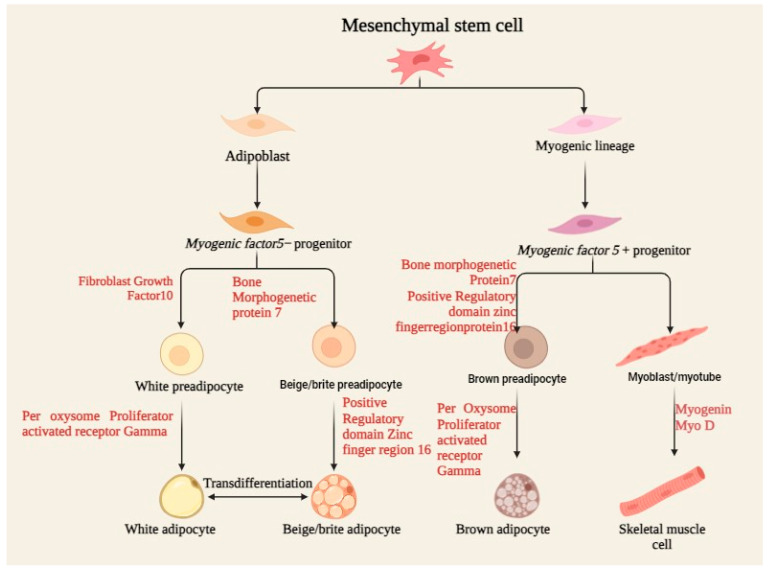
Process of MSC differentiation, including osteoblasts, chondrocytes, and adipocytes, under specific biochemical and mechanical cues, supporting tissue regeneration and repair.

Mesenchymal stem cells (MSCs) are multipotent stromal cells that differentiate into a variety of cell types including osteoblasts, chondrocytes, and neurons, as demonstrated in Figure 2. The differentiation capacity of MSCs is regulated by several biochemical and biophysical parameters, often acting in concert to guide cell fate. This review discusses how several environmental factors interact with each other to modulate MSCs’ differentiation capacity. The surface shape at the nanoscale, together with immobilized growth factors, can also synergistically influence MSC differentiation. TiO_2_ nanotubes of varying diameters, when coated with bone morphogenetic protein-2 (BMP-2), have differential impacts on MSC differentiation. A BMP-2 coating on 100 nm nanotubes promotes chondrogenic differentiation, whereas 15 nm nanotubes stimulate osteogenic differentiation. This implies that the lateral nanoscale spacing of BMP-2 provides environmental cues modulating lineage-specific differentiation and cell survival [32]. The combination of biochemical and structural cues in a 3D-printed matrix can direct MSC development for targeted tissue regeneration.

### 4.3. Migration and Homing

MSC homing is a multistep process involving numerous molecular interactions. The interaction between MSCs and the endothelium involves, first, the use of selectins, followed by an activation event with cytokines, arresting by integrins, and finally passing across the endothelial barrier through the action of matrix remodelers, after which the cells extra vascularly migrate into chemokine gradients [33]. Key signaling pathways implicated in MSC homing include the PI3K-Akt, MAPK, and Jak/Stat pathways, which are activated in response to chemokines such as stromal cell-derived factor-1 (SDF-1) [34]. Several ways have been tested to improve MSC homing efficiency. Genetic manipulation of MSCs to overexpress particular factors, such as fibroblast growth factor 21 (FGF21), has been found to improve their migratory potential and homing to injured regions [35]. Cell surface modification and the use of biomaterial carriers are other important factors in regulating MSC migration.

### 4.4. Paracrine Effect

Mesenchymal stem cells are well known for their strong regeneration capabilities, mainly attributed to the effects of paracrine activities. These are widely reported in the production of a wide spectrum of bioactive chemicals affecting the behavior of cells and tissues around them towards healing and regeneration. Recently, mounting interest can also be seen in the literature on the role of the microenvironment and biomaterials in modifying these paracrine effects through the improvement of MSCs’ therapeutic potential.

It has been shown that the enhancement of cell–cell contact by biomaterials improves the paracrine activity of MSCs. Thus, inhibition of N-cadherin significantly reduces the paracrine effects of MSCs, apparently showing that cell–cell contacts could modify the activity of MSCs [36]. Similarly, research on low-temperature-printed hierarchical porous sponges found that these scaffolds improve MSC adherence, retention, and survival, enhancing cell–material interactions. The synergetic effects of MSCs are described in Table 2. MSCs cultivated on these sponges demonstrated dramatically better paracrine activities, including increased production of immunomodulatory, angiogenic, and osteogenic components. MSCs’ paracrine actions play an important role in a variety of regeneration processes. MSC paracrine effects are regulated through complicated interactions with the extracellular matrix, adjacent cells, and soluble substances [37].

## 5. Synergistic Effects on H_2_O_2_ and CAP

### 5.1. Molecular Mechanism of Interaction

The synergistic effects of hydrogen peroxide (H_2_O_2_) and cold atmospheric plasma (CAP) have received a lot of attention because of their potential uses in medical and microbiological disinfection. This review seeks to explain the molecular mechanisms underlying the interaction of H_2_O_2_ and CAP, with an emphasis on their combined effects on tumor cells and microbial disinfection. The combination of H_2_O_2_ and nitrite, both long-lived species in CAP, is critical in causing selective apoptosis in tumor cells. The reaction between H_2_O_2_ and nitrite produces peroxynitrite, which is the first stage of the process. This peroxynitrite subsequently reacts with leftover H_2_O_2_ to produce singlet oxygen, which deactivates catalase molecules on the surface of tumor cells. The inactivation of catalase is significant because it allows H_2_O_2_ and peroxynitrite, which are normally destroyed by catalase, to remain at the inactivation site. This persistence causes the creation of secondary singlet oxygen, which further inactivates catalase and creates a self-sustaining cycle of singlet oxygen generation and catalase inactivation.

Catalase inactivation promotes the entry of H_2_O_2_ through aquaporins, depleting intracellular glutathione and sensitizing cells to death by lipid peroxidation. Furthermore, this mechanism creates intercellular apoptosis-inducing HOCl signaling, which is initiated by active NOX1 and completed by lipid peroxidation via hydroxyl radicals, activating the mitochondrial pathway of apoptosis. This model describes the selective impact of CAP and plasma-activated medium (PAM) towards tumor cells, contrary to prior theories that stated ROS/RNS from CAP or PAM were sufficient to directly cause cell death in tumor cells [44].

### 5.2. Complementary Antioxidant Pathways

Several studies have shown that when multiple chemicals are mixed, they provide synergistic antioxidant effects. For example, combining quercetin and catechin has been found to greatly increase antioxidant activity in H_2_O_2_-stimulated HepG2 cells. This synergism is mediated by the Keap1–Nrf2 signaling pathway, with BACH1 acting as a negative regulator. The presence of quercetin and catechin stimulates the upregulation of let-7a-5p and miR-25-3p, which in turn downregulate BACH1, resulting in reduced reactive oxygen species (ROS) formation and increased cell proliferation [45]. Similarly, phenolic acids and carotenes have been shown to have synergistic antioxidant effects in H_2_O_2_-induced H9c2 cells. Phenolic acids promote carotenoid absorption and membrane transporter expression, boosting the antioxidant response. A combination of β-carotene and caffeic acid reduced intracellular ROS levels and boosted nuclear Nrf2, indicating a synergistic impact through the activation of carotene membrane transporters by phenolic acids [46]. Cold atmospheric plasma (CAP) and plasma-activated media (PAM) have been found to have synergistic effects on tumor cells. The interaction of nitrite and H_2_O_2_, two long-lived molecules in CAP, results in the synthesis of peroxynitrite and then singlet oxygen generation. This process disables catalase on the surface of tumor cells, allowing H_2_O_2_ and peroxynitrite to accumulate and cause death via lipid peroxidation and the mitochondrial route. This concept emphasizes the specific impact of CAP and PAM on tumor cells, mediated by their own ROS and reactive nitrogen species (RNS) [44].

### 5.3. Enhancement of Cell Survival

Preconditioning cells with low levels of H_2_O_2_ has been found to improve their resistance to oxidative stress. H_2_O_2_ preconditioning improves survival in human adipose-derived stem cells (hASCs) by lowering intracellular ROS levels by upregulating the transcription factor Nrf2 and its associated antioxidant enzymes. Furthermore, it decreases the release of pro-inflammatory chemicals and adjusts the cellular metabolism to meet the metabolic needs required for survival under oxidative circumstances [47].

### 5.4. Optimization and Treatment Parameters

The maximal intracellular H_2_O_2_ concentration can be used to quantify cell susceptibility toward exogenous H_2_O_2_ in cold atmospheric plasma and plasma-treated liquids for cancer treatment [48]. Combining cold atmospheric pressure plasma jet (CAP) with hydrogen peroxide (H_2_O_2_) provides dramatic synergistic effects in bacterial disinfection through enhanced membrane transportation of reactive species and oxidation of intracellular molecules [49]. Singlet oxygen from cold atmospheric plasma (CAP) or plasma-activated medium (PAM) triggers tumor cells to generate high concentrations of secondary singlet oxygen, inactivating their protective catalase and reactivating ROS/RNS-dependent apoptosis-inducing signaling [50]. H_2_O_2_ surface treatment improves the surface properties and biological performance of calcium phosphates, enhancing ROS generation and cytocompatibility [51]. A combination of cisplatin and cold atmospheric plasma treatment shows a synergistic anticancer effect with low cytotoxicity against normal cells [52]. A H_2_ self-generation nano-platform strengthens the curative effect of chemodynamic therapy and enhances the Fenton reaction rate, enhancing multimodal synergetic therapy [53].

## 6. Technical Considerations and Optimization

One of the most promising things in regenerative medicine is MSCs, which are capable of differentiation into a variety of tissue types, and which play immune-modulatory roles. The technical aspects of optimizing MSC therapy involve multiple facets, such as the selection of sources, from bone marrow, adipose tissue, and umbilical cord, and using efficient isolation techniques like flow cytometry or MACS to ensure that the population obtained is pure [41]. For quality control, appropriate characterization, including surface markers such as CD73, CD90, and CD105, as well as functional assays to evaluate differentiation capacity, must be considered [54]. Delivery modes—whether intravenous, intramuscular, or localized—must also allow for the retention of MSCs at the targeted site; this can be enhanced using biomaterials or genetic modification.

The survival and function of MSCs are dependent on the local microenvironment, particularly through factors such as hypoxia and inflammation. Rather, therapeutic efficacy is achieved through the paracrine mechanism whereby MSCs produce growth factors that trigger tissue repair [55]. The scope of genetic engineering alone enhances the potential for MSCs to target specific diseases or deliver therapeutic agents, while encapsulation in biomaterials protects and controls the delivery [56]. Further emerging technologies are nanotechnology and 3D printing, which are rapidly advancing in the field, primarily through the improvement of MSC delivery methods and tissue engineering. Other promising approaches include personalized medicine, where MSC therapy is matched to the genetic profile and particular characteristics of a disease for a patient.

Molecular hydrogen (H_2_) is gaining more and more interest as a therapeutic agent due to its strong antioxidant and anti-inflammatory effects, thus making it a potentially useful additive in MSC therapy. To enhance the therapeutic efficacy of molecular hydrogen in MSC applications, the delivery of molecular hydrogen needs to be optimized carefully. There are various ways through which molecular hydrogen can be delivered [57]. Each of them has its advantages and disadvantages. Hydrogen-rich water is another handy, non-invasive delivery system; however, hydrogen loss is one of the major challenges for the storage and consumption of water. The studies [58] validate its protective role against oxidative stress-mediated damage. In contrast, injection methods, while providing site-specific delivery, involve risks of tissue damage and embolism, as reported by studies like [59].

In addition to that, enhancements in hydrogen generation and storage systems in terms of electrolysis and metal–acid reactions are included, with the problems noted regarding both efficiency and purity issues revealed in past studies. The design of hydrogen delivery devices should emphasize portability, ease of use, and compatibility with various delivery methods, with biocompatibility serving as a crucial factor, as evidenced by studies such as [60]. Through better development of delivery systems, researchers may further the therapeutic benefits of MSC therapy, especially when applied in diseases where molecular hydrogen holds beneficial pro-anti-inflammatory and regenerative actions.

Cold atmospheric plasma (CAP) is a kind of non-thermal plasma that is generated at atmospheric pressure, characterized by the presence of reactive oxygen and nitrogen species (ROS and RNS), charged particles, and ultraviolet radiation. Such properties might make CAP a potential therapeutic tool for biomedical applications specifically for MSCs. The possible application of CAP for MSC therapy becomes meaningful only in the case of adjustment and optimization of several technical conditions. Among these CAP devices are atmospheric pressure plasma jets (APPJs), in which dielectric barrier discharges can generate homogeneous plasma and work at high values of output power while being very sensitive to arcing, and which possess a complicated design [61,62]. Gliding arcs provide a deep penetration compared with plasma density but are dangerous and susceptible to thermal damage [63].

Other important factors in CAP optimization for MSC therapy are treatment parameters. These include plasma exposure time, power, and gas composition. Studies assert that optimal plasma exposure times depend on the biological effects intended and the type of MSCs [64]. The influence of plasma power on MSC viability, proliferation, and differentiation is significant, with research indicating that lower power levels are typically optimal for maintaining cell function [65]. Furthermore, the composition of gases can be customized to provoke particular responses in MSCs, thereby enhancing processes such as wound healing [66]. Combination therapies, for instance, can also be utilized to further enhance the potential of CAP by leveraging synergistic effects with growth factors for tissue regeneration. In conclusion, optimizing the configurations of CAP devices, treatment parameters, and cell culture conditions can empower CAP as an effective tool for augmenting MSC therapy and improving regenerative outcomes.

### Timing and Duration of Molecular Hydrogen and Cold Atmospheric Plasma in Enhancing Mesenchymal Stem Cell Therapy

The timing and duration of treatments utilizing molecular hydrogen (H_2_) and cold atmospheric plasma (CAP) are highly critical and, in themselves, can significantly impact the effectiveness of mesenchymal stem cell therapy. Preconditioning MSCs with H_2_ before injection has been established to enhance their survival, migration, and differentiation capacity. Simultaneous administration of H_2_ during MSC therapy enhanced engraftment and therapeutic outcomes. The period of H_2_ treatment depends on the disease condition to be treated: short-term H_2_ treatment might be enough for acute diseases, whereas long-term H_2_ treatment is necessary for chronic diseases. However, the duration depends on the disease severity, response from the patient, and potential side effects [67]. In the same way, optimal timing of CAP treatment could prepare tissues before MSC application, enhance engraftment during treatment, and facilitate tissue regeneration afterwards through MSC-based therapies. The treatment period with CAP shares a similar process: temporary therapy with H_2_ is successful in treating acute diseases, while chronic diseases require treatment over a longer time. In the case of H_2_ as well as CAP treatment, it is crucial to have quality control. In H_2_, gas purity, testing the concentration of hydrogen, and stability are of utmost importance [68]. CAP quality control overlaps plasma parameters like power and gas composition together with uniformity, with safety measures directed toward ensuring proper functioning of the device as well as the efficiency of the biological effects produced [69]. Optimization of the timing, duration, and quality control of both H_2_ and CAP treatments can significantly improve the efficacy of MSC therapies for several clinical applications.

In short, MSC therapy optimization indeed requires careful choices among the sources of MSCs, delivery modalities, and advanced technologies such as genetic engineering, nanotechnology, and personalized medicine.

## 7. Clinical Applications and Future Perspectives

### 7.1. Current Clinical Status

The contemporary clinical status of molecular hydrogen and cold atmospheric plasma in enhancing MSC therapy is optimistic yet remains in its infancy. The antioxidant and anti-inflammatory properties of H_2_ were recently highlighted by studies undertaken in [70], thereby adding power to MSCs’ survival, migration, and differentiation capability. Thus, they may unlock new treatment therapies for ischemic heart disease, organ failure, or diseases stemming from inflammation. Nonetheless, there remains a necessity for large-scale randomized trials, particularly due to challenges related to standardized delivery methods and dosimetry. A review [71] highlighted the growing interest in H_2_ for stem cell therapies; however, it underscored the importance of obtaining more substantial clinical evidence to verify its efficacy and safety.

The therapeutic application of H_2_ has been tested in numerous clinical trials, but its administration has been evaluated for multiple diseases, such as stroke, diabetes, and inflammatory bowel disease. Still, a very limited number of reports exist showing specific data on the combination of H_2_ and MSC treatment. Large-scale randomized controlled trials are required to determine the clinical efficacy of this combination. In some experiments, it has been demonstrated that molecular hydrogen may help with oxidative stress and inflammation, which are different indicators of MSCs’ therapeutic potential. Studies such as [72] present an increasing interest in H_2_ due to its use as a medical gas. Still, there remain many challenges, such as the variability in delivery methods, like inhalation and hydrogen-rich water, and dosage, making it complex to standardize H_2_ therapy across various clinical settings. There is also the lack of a standardized procedure for the accurate quantification of hydrogen concentration in tissues, limiting the ability to tailor treatments.

Table 3 represents the many different clinical trials for CAP and molecular hydrogen indicating favorable effects, especially within applications related to wound healing and other dermatological issues. When applying CAP with MSCs, the latter’s ability to produce ROS/RNS properties has been advantageous, although there are relatively few clinical trials that focus solely on the issue of MSC therapy. Some of those studies demonstrated that CAP treatment increased the regenerative capacity of MSCs, which means that it increased their effectiveness in tissue repair [73,74]. Along with clinical applications, safety issues for patients have posed challenges regarding long-term exposure. On the other hand, the vast variability of configurations and dosimetry in CAP devices and the lack of uniform treatment parameters have hindered the standardization of therapy. More studies need to be conducted to strengthen these technical aspects further.

### 7.2. Potential Therapeutic Applications

Molecular hydrogen (H_2_) and cold atmospheric plasma (CAP) now represent advanced agents that show much promise for improving MSC therapy. H_2_ and CAP enable cell and tissue biochemical features to be selectively modulated by a unique combination of the potent antioxidant and anti-inflammatory effects of H_2_ and by changes in the behavior of cells modified by CAP with improved tissue regeneration. This has been shown to improve the therapeutic potential of MSCs through their antioxidant and anti-inflammatory properties. Oxidative stress and modulated immune responses that will contribute to the survival, migration, and differentiation of MSCs are reduced by molecular hydrogen and form a favorable microenvironment for effective stem cell therapy.

**Figure 3:**
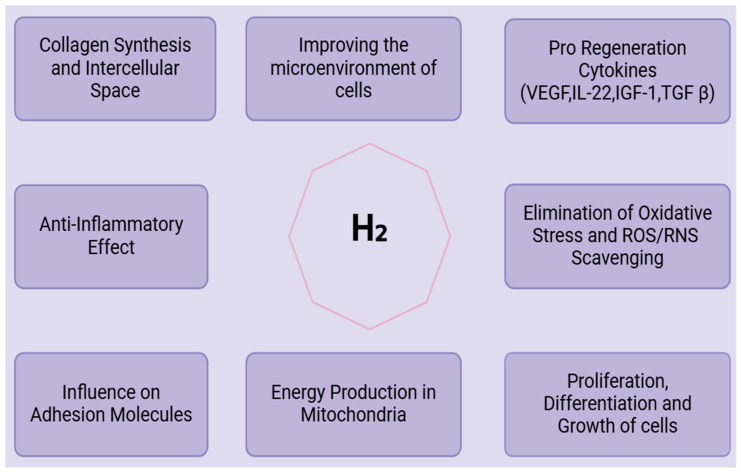
Clinical applications of molecular hydrogen.

Molecule hydrogen has been considered an agent for enhancing results in ischemic conditions, in which tissue damage arises due to a low supply of blood. Figure 3 represents the therapeutic applications of molecular hydrogen. The antioxidant activity of H_2_ helps reverse oxidative injury due to reperfusion injury following ischemia. In stroke, H_2_ enhanced the therapeutic effectiveness of MSCs in ischemic stroke by improving the survival and repair of cells and neurons [77]. In the case of myocardial infarction, H_2_ can protect heart tissue from ischemia–reperfusion injury, and thus has the potential to improve cardiac function in patients after a heart attack. In peripheral artery disease and other similar diseases, H_2_ protects tissues from ischemic injury through its modulative action on oxidative stress and inflammation. Hydrogen molecules have neuroprotective effects and seem, thus, promising adjuncts in MSC therapy against neurodegenerative diseases, being capable of diminishing oxidative stress and inflammation in the brain and improving survival, as well as the differentiation capacity of MSCs into neural cells.

In the case of Alzheimer’s disease, there is evidence that H_2_ can prevent the oxidative stress associated with the disease and improve the preservation of cognitive functions. Studies on preclinical research have shown that H_2_ inhalation has provided neuroprotection to dopaminergic neurons and has delayed the worsening of Parkinson’s disease. H_2_, for its anti-inflammatory properties, is beneficial in inflammatory diseases [79]. By reducing inflammation and oxidative damage, H_2_ enhances the ability of MSCs to repair and regenerate tissues in conditions characterized by chronic inflammation. H_2_ could diminish the symptoms of rheumatoid arthritis as it suppresses pro-inflammatory immune responses, thus enhancing the ability of MSCs to repair and regenerate damaged cartilage. H_2_, in a preclinical model of colitis, was shown to exert positive effects through the reduction of intestinal inflammation [80]. The survival and migration of MSCs have shown great promise in improving molecular hydrogen’s healing of wounds. It can improve the healing of challenging wounds such as diabetic ulcers, burns, and pressure ulcers [80], as illustrated in Figure 3.

**Figure 4:**
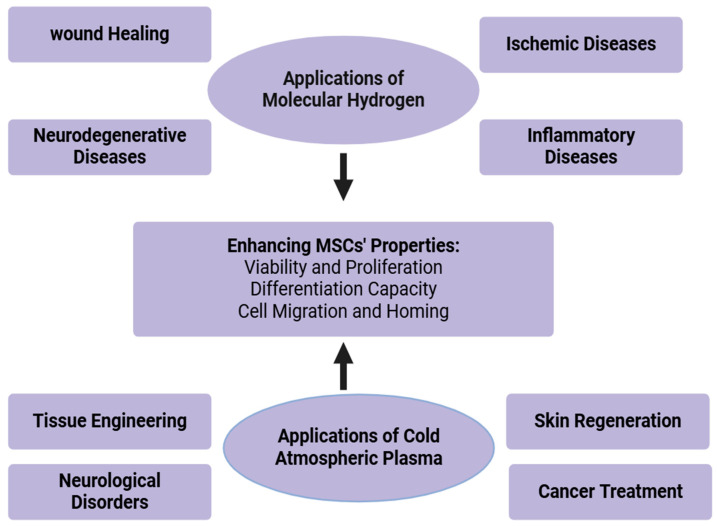
Therapeutic applications of molecular hydrogen and cold atmospheric plasma.

Cold atmospheric plasma is a partially ionized gas, producing reactive oxygen and nitrogen species (RONS) that are known to become involved in cell functions such as proliferation, migration, and differentiation. CAP has great potential in the use of MSC therapy to combat treatments for wound healing, cancer, and other clinical applications. CAP has been extensively studied in enhancing tissue regeneration and promoting chronic wound healing. The synergistic effects of CAP in combination with MSC therapy seem to have superiority in cellular proliferation and tissue repair. CAP, in combination with MSCs, would accelerate the healing of diabetic ulcers by accelerating angiogenesis and having lower risks of infection [76]. CAP also has potent activity on skin conditions such as dermatitis, psoriasis, and acne. By its microbicidal action, it can also suppress inflammation and facilitate the regeneration of skin, so it is one useful reagent in many dermatologic treatments [81]. CAP has been shown to possess potential as an adjunct in the treatment of cancers because it could mediate the selective killing of cancerous cells and can also enhance the effects of chemotherapy and radiotherapy [82]. The combination of molecular hydrogen and cold atmospheric plasma in MSC therapy presents synergistic effects, enhancing the overall therapeutic potential of MSCs. This duality may improve cell proliferation, migration, differentiation, and survival altogether, leading to better outcomes in all conceivable therapeutic applications [78]. However, future research should be dedicated to standardizing treatment protocols, optimizing dosages, and ensuring long-term safety in clinical settings. Applications are illustrated in Figure 4.

### 7.3. Potential Challenges with Solutions for Combining Molecular Hydrogen and Cold Atmospheric Plasma in Mesenchymal Stem Cell Therapy

The combination of molecular hydrogen, H_2_, with cold atmospheric plasma, CAP, in MSC therapy is intricate and throws up many challenges that need due contemplation. Some challenges include the necessity of optimizing dosages to the regulatory issues regarding using such therapies. The optimal dose for the pre-treatment of MSCs using molecular hydrogen is not clear; an extended exposure might be harmful due to side effects. Optimal dosing with molecular hydrogen should be established through extensive preclinical and clinical studies [83]. Long-term risks should be evaluated using longer follow-up studies. Marks of oxidative stress and inflammation should be continuously monitored for safety. The failure to standardize protocols in applying molecular hydrogen and CAP across studies and clinical settings brings about variable outcomes. This would involve coordination among research institutions, industry, and bodies of regulation to formulate uniform procedures for applying H_2_ and CAP [84]. The production and application of molecular hydrogen and CAP are expensive. This shall set a cost to patients wanting to undergo such therapies. Investment in research to improve the cost-efficiency of producing hydrogen and CAP devices should involve partnership with industries to help scale up their production and distribution [84]. The issue with MSCs is that they can induce a perverse immune response leading to rejection. Molecular hydrogen and CAP need to be optimized regarding their immunomodulatory properties to decrease this chance.

Combination therapies with immunosuppressive agents or genetic modification of MSCs to reduce their immunogenicity would counter rejection. Preclinical studies should evaluate whether H_2_ and CAP can drive MSC immune evasion [85]. The technical challenge lies in the fact that it is hard to manufacture and install CAP to such high precision. Inconsistency in the outcome arises due to heterogeneous devices that have been developed and treatment parameters [84]. The proper orientation of healthcare workers about using devices appropriately will ensure consistent and reproducible treatments. The radicals produced by CAP are also good but, if not controlled, may cause oxidative stress and cellular injury [44]. CAP can easily manipulate proliferation and differentiation cell fates, which in the wrong sites could lead to undesirable therapeutic outcomes [65]. The interaction between molecular hydrogen and CAP might fail to result in an optimal effect if it is not orchestrated. The synergy effects of H_2_ and CAP should be preclinically examined. Studies on the time, sequence, and dose–response will indicate the best possible time when treatment is given, so that maximum benefit may be achieved [44]. The use of molecular hydrogen as part of MSC treatment with CAP brings additional challenges in the ethical and regulatory senses. The regulatory bodies and the ethical issues will be concerned about the consent and safety of the patient, before the administration of treatment [67]. Addressing these challenges with collaborative research, technological breakthroughs, and careful regulatory planning will eventually lead to the successful integration of molecular hydrogen and CAP in mesenchymal stem cell therapy.

## 8. Conclusions

In conjunction with the introduction of molecular hydrogen (H_2_) and cold atmospheric plasma into MSC therapy, several promising research directions can be drawn forth that may considerably enhance the therapeutic efficacy of MSCs. For starters, it is deemed crucial to understand the mechanisms at the molecular basis of the effects of H_2_ and CAP on MSCs. Further investigation of specific molecular pathways will elaborate on how such agents may alter cellular function in the context of proliferation, differentiation, and tissue regeneration. The research has shown that this improves cell survival and differentiation in MSC therapy. Moreover, the simultaneous use of H_2_ with growth factors can likely prove to optimize MSC therapy for various purposes, as shown by [46]. This may pave the way for personalized medicine, tailoring treatments based on the characteristics and responses of patients, such as the example shown in [25]. Follow-up studies for long periods are essential to evaluate the longevity and sustainability of these therapeutic effects. Research work such as [66] will help confirm and establish the efficacy and safety of MSC therapies based on their H_2_ and CAP enhancement, which is fundamental for clinical translation. Lastly, the advanced technology associated with CAP devices can further improve the precision and effectiveness of the treatment by doing so through discussions presented as in [68]. Similarly, the nanoparticle-based delivery systems targeted to H_2_ therapy studies, such as [86], may potentially promote advancing the therapeutic response with minimal adverse effects.

## Figures and Tables

**Table 1:** Comparison of the properties and applications H_2_ and CAP.

	Molecular Hydrogen (H_2_)	Cold Atmospheric Plasma (CAP)
Properties	Small, neutral molecule - High diffusibility, penetrating cellular membranes - Antioxidant, anti-inflammatory	Ionized gas is composed of ions, electrons, and radicals - Generates reactive species (e.g., ROS and RNS) - Non-thermal
Mechanism of action	Scavenges hydroxyl radicals - Inhibits inflammatory cytokines - Protects mitochondrial function	Induces oxidative stress in a controlled manner - Alters cell membrane potential - Modulates redox signaling
Stem cell proliferation	Enhances MSC proliferation - Improves MSC viability	Can enhance or inhibit MSC proliferation depending on dose and duration
Stem cell differentiation	Promotes osteogenic, chondrogenic, and adipogenic differentiation	Induces osteogenic differentiation - Potential to modulate other differentiation pathways based on ROS levels
Anti-inflammatory effects	Reduces pro-inflammatory cytokine expression - Beneficial for inflammatory-related stem cell therapies	Decreases pro-inflammatory responses in MSCs under specific conditions - Supports wound healing applications
Oxidative stress tolerance	- Reduces ROS damage in MSCs - Enhances MSC tolerance to oxidative stress	Controlled ROS generation can promote cellular adaptation - Excessive ROS may be cytotoxic, requiring optimization
Applications in MSC therapy	Treatment for oxidative stress-related diseases - Promotes tissue regeneration - May enhance MSC therapy efficacy in neuroprotection, cartilage repair, and other regenerative applications	Used for wound healing and anti-cancer therapies - Enhances MSC-mediated tissue repair and regeneration - Potential use in skin rejuvenation, anti-inflammation, and infection control
Safety and side effects	- Generally safe with low toxicity - Minimal side effects reported	- Safe under controlled conditions - Potential cytotoxicity at higher doses due to ROS production

**Table 2:** Demonstration of synergetic effects on MSC biology.

Category	Synergetic Effect	References
Cell survival and proliferation	The non-cellular microenvironment affects the proliferation, survival, and differentiation of MSCs. Matrix topology and cell shape guide the differentiation: spread cells become differentiated as osteocytes, but spherical morphology will generate adipocytes. Matrix rigidity and mechanical feedback influence differentiation.	[28,31,38,39].
Differentiation capacity	Targeted differentiation via biochemical factors and mechanical cues: FSS and matrix stiffness. Structure of nanotubes and growth factor BMP-2 influence lineage-specific differentiation. It was also demonstrated that co-cultures with osteocytes enhance MSC osteogenesis.	[32,40,41].
Migration and homing	The key signaling pathways PI3K-Akt, MAPK, and Jak/Stat mediate homing. Genetic manipulation (FGF21) and surface modifications enhance MSC migration. The functionalized ECM microcarrier systems aid in improving the adhesion and migration functions of MSCs.	[33,35,42].
Paracrine effect	Macroporous scaffolds result in increased secretion, hence ensuring effective tissue regeneration. Increased MSC secretions and improvements in ischemic injury models are due to biomaterial interaction and increased FAK activation.	[36,37,43].

**Table 3:** Clinical trials and outcomes of molecular hydrogen and cold atmospheric plasma.

Study	MSC Type	Target Site	Outcomes	Clinical Trial Detail	References
Molecular hydrogen for osteoarthritis	bone marrow (BM-MSCs) osteoarthritis	Osteoarthritis	Greater cartilage repair and reduced oxidative stress and inflammation	Phase II trial: MSC-treated patients combined with molecular hydrogen had a significantly greater range of motion in the joints and less pain.	[75]
Cold atmospheric plasma for wound healing	Adipose-derived MSCs (AD-MSCs)	Diabetic ulcers	Quick healing, enhanced MSC proliferation, and differentiation towards keratinocytes	Randomized controlled trial: CAP-treated MSCs were shown to have significantly improved healing rates when compared to the MSC control group.	[76]
Molecular hydrogen in ischemic stroke	Umbilical cord-derived MSCs (UC-MSCs)	Ischemic stroke	Reduced infarct size, increased neuroprotection, and favorable neurological outcomes	Phase I trial: adjunct therapy demonstrated neuroprotection with reduced oxidative stress	[77]
CAP for spinal cord injury	Bone marrow-derived MSCs	Spinal cord injury	Promoting MSC viability, migration, and differentiation into neural cells	Preclinical study: CAP-treated MSCs have promising regenerative potential to improve motor function	[78]
Hydrogen gas in cardiac regeneration	Cardiac-derived MSCs	Myocardial infarction	Restored cardiac function, decreased fibrosis, and enhanced angiogenesis	Phase II trial hydrogen gas inhalation after MSC treatment improved left ventricular function.	[79]
